# Amygdalin as a Promising Anticancer Agent: Molecular Mechanisms and Future Perspectives for the Development of New Nanoformulations for Its Delivery

**DOI:** 10.3390/ijms241814270

**Published:** 2023-09-19

**Authors:** Maria Spanoudaki, Sofia Stoumpou, Sousana K. Papadopoulou, Dimitra Karafyllaki, Evangelos Solovos, Konstantinos Papadopoulos, Anastasia Giannakoula, Constantinos Giaginis

**Affiliations:** 1 Department of Nutritional Sciences and Dietetics, School of Health Sciences, International Hellenic University, 54700 Sindos, Greece; maryspan1@gmail.com (M.S.); stoumpous@gmail.com (S.S.); souzpapa@gmail.com (S.K.P.); agianakoula@ihu.gr (A.G.); 2 Clinical Dietetics and Nutritional Department, 424 General Military Hospital, 56429 Thessaloniki, Greece; 3 Department of Nutrition and Dietetics, School of Physical Education, Sport Science and Dietetics, University of Thessaly, 42132 Trikala, Greece; dkarafyllaki@uth.gr; 4 Orthopedic Department, 424 General Military Hospital, 56429 Thessaloniki, Greece; e.m.solovos@army.gr (E.S.); k.a.papadopoulos@army.gr (K.P.); 5 Laboratory of Plant Physiology and Postharvest Physiology of Fruits, Department of Agriculture, International Hellenic University, 54700 Sindos, Greece; 6 Department of Food Science and Nutrition, School of Environment, University of Aegean, 81400 Lemnos, Greece

**Keywords:** amygdalin, laetrile, vitamin B17, anticancer effects, anticancer molecular mechanisms, cancer, apoptosis, cancer cell proliferation, nanoparticles, nutritional supplements, drug discovery

## Abstract

Cancer rates are increasing, and cancer is one of the main causes of death worldwide. Amygdalin, also known as vitamin B17 (and laetrile, a synthetic compound), is a cyanogenic glycoside compound that is mainly found in the kernels and pulps of fruits. This compound has been proposed for decades as a promising naturally occurring substance which may provide anticancer effects. This is a comprehensive review which critically summarizes and scrutinizes the available studies exploring the anticancer effect of amygdalin, highlighting its potential anticancer molecular mechanisms as well as the need for a nontoxic formulation of this substance. In-depth research was performed using the most accurate scientific databases, e.g., PubMed, Cochrane, Embase, Medline, Scopus, and Web of Science, applying effective, characteristic, and relevant keywords. There are several pieces of evidence to support the idea that amygdalin can exert anticancer effects against lung, breast, prostate, colorectal, cervical, and gastrointestinal cancers. Amygdalin has been reported to induce apoptosis of cancer cells, inhibiting cancer cells’ proliferation and slowing down tumor metastatic spread. However, only a few studies have been performed in in vivo animal models, while clinical studies remain even more scarce. The current evidence cannot support a recommendation of the use of nutritional supplements with amygdalin due to its cyano-moiety which exerts adverse side effects. Preliminary data have shown that the use of nanoparticles may be a promising alternative to enhance the anticancer effects of amygdalin while simultaneously reducing its adverse side effects. Amygdalin seems to be a promising naturally occurring agent against cancer disease development and progression. However, there is a strong demand for in vivo animal studies as well as human clinical studies to explore the potential prevention and/or treatment efficiency of amygdalin against cancer. Moreover, amygdalin could be used as a lead compound by effectively applying recent developments in drug discovery processes.

## 1. Introduction

Characterizing global variations in disease burden and trends over time provides important information on the etiology of cancer and serves as a basis for prevention strategies and treatment. Tumor malignancies are considered a leading cause of death worldwide; the number of new cancer cases globally has reached 19.3 million, and nearly 10 million people died in 2020 [1]. Alarmingly, updated evidence estimates that the number of new cases from 2020 to 2040 will be 28.4 million worldwide [2]. In addition, the most recent World Health Organization (WHO) data show that breast cancer remains the most prevalent malignancy in women in 158 out of 183 countries (86%) and the leading cause of cancer-related mortality in 173 out of 183 countries (95%) [3]. The second most common cause of cancer death worldwide and the third most prevalent malignancy in terms of diagnosis is colorectal cancer, based on the Global Cancer Statistics 2020 (GLOBOCAN) [1,4]. Moreover, current mortality patterns and longitudinal trends for the major cancer types in 47 countries worldwide, excluding Africa, show that the rates of infection-related cancers, such as cervical and stomach cancer, and tobacco-related cancers, such as lung and esophageal cancers, have increased approximately 10-fold [5]. In the European Union (EU), the total projected number of cancer deaths in 2023 is estimated to be 1,261,990 (702,214 for men and 559,776 for women) [6]. Total cancer mortality in 2022 was estimated at 1,269,200 [7], while colorectal and lung cancers were the leading causes of cancer deaths (>30%) in the EU. In Greece, the number of new cases in 2020 was 64,530, while the number of deaths was 33,166 [5,6,7]. In the United States, the number of emerging cancer cases in 2023 will be approximately 1,958,310, or approximately 5370 cases per day [8]. In this context, it should be noted that these estimated cases for 2023 are based on recently available incidence data up to 2019 and do not consider the impact of the COVID-19 pandemic on cancer diagnoses [8]. Accordingly, the projected cancer deaths in 2023 are based on data up to 2020 [8].

A topic of interest is the role of amygdalin, also known as vitamin B17 or laetrile (its synthetic compound), in the prevention and/or co-treatment of cancer. Several studies have demonstrated a wide range of biological properties for amygdalin, suggesting that it may exert a preventive or even a co-treatment impact on cervical, breast, prostate, lung, and bladder cancers, which may mainly be ascribed to the inhibition of cancer cell proliferation [9,10,11,12,13]. Both in vitro and in vivo evaluations of the biological actions of amygdalin extracts from three cassava (*Manihot esculenta*) cultivars grown in Benin demonstrated that this naturally occurring molecule may act effectively in cancer prevention and co-treatment by suppressing the formation of cancer cells [14]. In vitro studies have documented the induction of apoptosis by amygdalin because of the increased expression of Bax protein and caspase-3 and decreased expression of the anti-apoptotic protein Bcl-2 [15,16]. In the field of the chemopreventive potential of amygdalin, Erikel et al. (2023) noted that amygdalin may exert a modulatory effect on chemotherapeutic agents that seem to induce genomic damage in human lymphocytes [17].

The basic anticancer molecular mechanisms of amygdalin have mainly been ascribed to cell cycle inhibition, apoptosis induction, cytotoxic effect stimulation, and immune function regulation within the human body [15,18,19]. More to the point, the main molecular mechanism of apoptosis is the activation of the caspase-3 protease, which is initiated by cellular replication of the Bax protein of cytochrome C [20]. High expression of the pro-apoptotic protein Bax has been associated with apoptosis and subsequent cell proliferation [21]. In this regard, amygdalin has been considered to induce apoptosis by increasing the activity of caspase-3 in HeLa cells and downregulating Bcl-2 [22]. In parallel, Bax has appeared to be upregulated in HeLa cells treated with amygdalin, suggesting that an endogenous pathway may be involved in apoptosis [22]. Several human cell lines, including those from cancer cells of the lung, breast, colon, testes, prostate, rectum, and bladder, have shown that amygdalin can cause apoptosis and cell cycle arrest [19,20,22,23,24,25,26].

Despite the promising results from the currently available studies investigating amygdalin’s anticancer effects, there is a strong demand for further research in this topic. To date, the Food and Drug Administration (FDA) has not approved the medical use of amygdalin in the co-treatment of cancer or other medical conditions in the United States because of the lack of adequate data on the effectiveness of amygdalin and the risk of potential adverse side effects [27,28]. In this regard, the present review aimed to critically summarize and scrutinize the available data concerning the potential anticancer effects of amygdalin, highlighting its anticancer molecular mechanisms and its possible future application in clinical practice against cancer.

## 2. Methods

A comprehensive and in-depth review was conducted using the most accurate scientific databases, e.g., PubMed, Scopus, Web of Science, and Google Scholar, applying effective, characteristic, and relevant keywords such as “amygdalin” or “laetrile” or “vitamin B17” and “toxicity”, “antitumor effects” or/and “cancer” and “molecular mechanisms”. Inclusion criteria were studies written in the English language, clinical human studies, in vitro and in vivo animal studies, and randomized clinical trials (RCTs). Gray literature, commentaries, editorials, letters to the editor, reviews, abstracts in conference proceedings, and articles in non-peer-reviewed journals were excluded from the final analysis. The search was supplemented by scanning the reference lists of relevant studies and manually searching key journals, commentaries, editorials, and abstracts in conference proceedings. The retrieved surveys were additionally comprehensively checked for related studies quoted in their text. 

**Figure 1:**
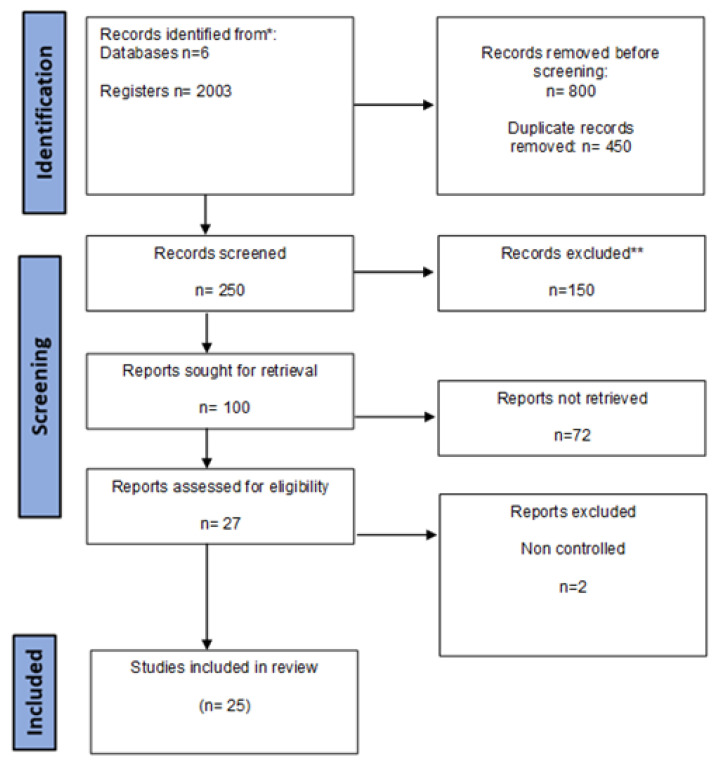
Flow chart diagram of the study selection. * The number of records identified from each database or register searched (rather than the total number across all databases/registers), ** How many records were excluded by a human and how many were excluded by automation tools.

All authors acted as reviewers. To enhance consistency among reviewers, all reviewers screened all the retrieved publications, discussed the results, and amended the screening and data extraction manual before beginning screening for this review. All reviewers worked in pairs to sequentially evaluate the titles, abstracts, and then full texts of all publications identified by our search for potentially relevant publications. We resolved disagreements on study selection and data extraction by consensus and discussion with all authors/reviewers, if needed. A data charting form was jointly developed by two reviewers (M.S. and C.G.) to determine which variables to extract. The two reviewers independently charted the data, discussed the results, and continuously updated the data-charting form in an iterative process. The findings were selected based on relevance, and the most relevant ones were chosen for discussion below according to the flow chart diagram depicted in Figure 1.

## 3. Results

### 3.1. Amygdalin: Basic Information and Properties

**Figure 2:**
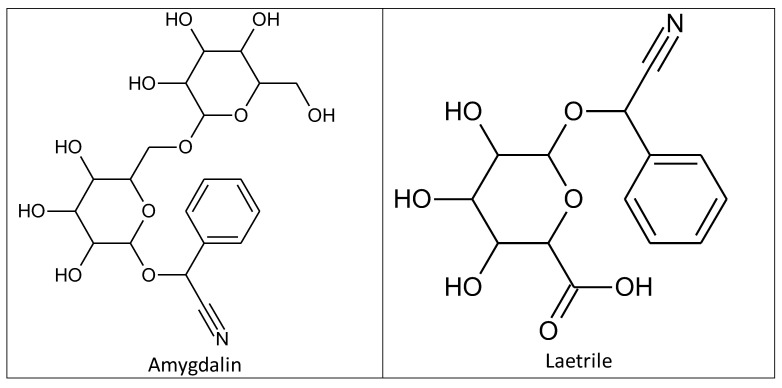
Chemical structures of amygdalin and laetrile.

Amygdalin was discovered in 1803 by Schrader in a study of bitter almond ingredients, and was first isolated in 1830 by two French chemists, Pierre-Jean Robiquet and Antoine François Boutron-Charlard [29,30]. Chemists Haworth and Wylam finally determined its exact chemical structure in 1923 (Figure 2) [31]. Amygdalin (d-mandelonitrile-β-d-glucoside-6-β-glucoside) is a cyanogenic glycoside compound which consists of dibenzaldehyde, hydrocyanic acid, and two glucose molecules (D-mandelonitrile-β-D-glucoside-6-β-glucoside) [32]. Its bioactive form (D-mandelonitrile-β-glucose) was used for a United States Patent (USP). Laetrile is a partly manmade, synthetic form of the natural substance amygdalin (Figure 2) [32]. In Mexico, the structure was differentiated and defined as D-mandelonitrile-β-gentiobioside [33]. The US National Center Institute (NCI) demonstrated that the Mexican form of amygdalin (oral and intravenous) did not comply with US drug standards and the substance was banned for human consumption [33]. Amygdalin is mainly found in the kernels and pulps of fruits such as plums, apricot pits, black cherries, peaches, and bitter almonds, and has been widely used in alternative medicine [34,35,36]. Amygdalin’s molecular formula is C20H27NO11, and its molecular weight is 457.42 g·mol^−1^ [37].

Amygdalin can produce mandelonitrile and pranazine through hydrolysis under the action of glucosidase, and is eventually broken down into hydrocyanic acid and benzaldehyde [30]. The cytotoxic effect of amygdalin on cancer cells in vitro and the distribution of amygdalin in plants that are commonly consumed in the human diet are two of the most compelling topics in recent research. However, it is not a new compound, and it has been used in traditional and alternative medicine for centuries due to its anticancer and anti-inflammatory properties and, in general, its numerous medical benefits [35,36,38]. It has been helpful in relieving pain and fever; suppressing coughs, thirst, and nausea; and as a cancer prevention and co-treatment agent in recent years [39,40]. 

### 3.2. Anticancer Effects and Molecular Mechanisms of Amygdalin: In Vitro and In Vivo Evidence

**Figure 3:**
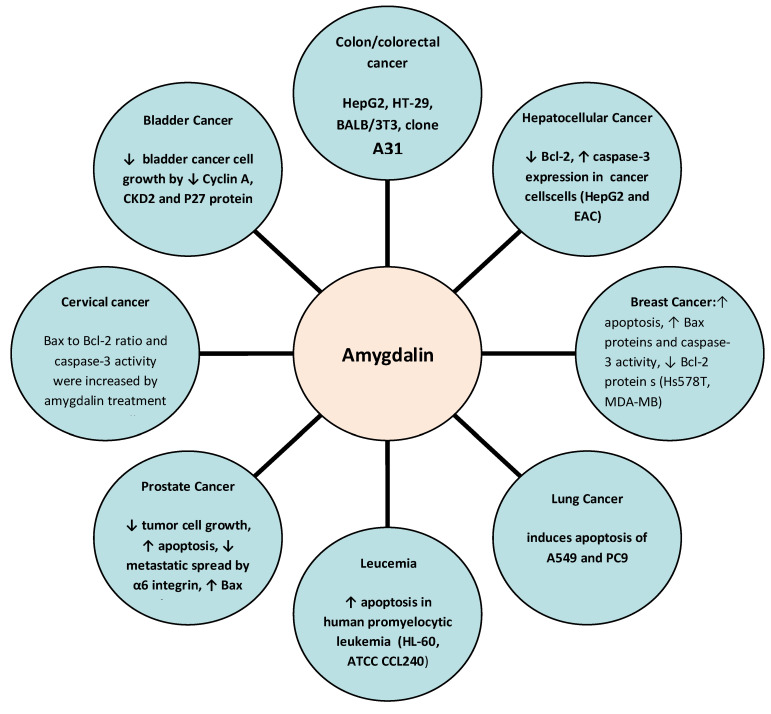
Anticancer molecular mechanisms of amygdalin. ↑: increase, ↓: decrease.

To date, several studies have explored the potential anticancer effects of amygdalin, highlighting its anticancer molecular mechanisms especially in lung, breast, prostate, colorectal, cervical, and gastrointestinal cancers. The potential anticancer effects and molecular mechanisms of amygdalin are depicted in Figure 3. Clinical studies in human subjects as well as in human and animal cancer cells are presented in Table 1.

#### 3.2.1. Lung Cancer

Amygdalin could be beneficial as a co-therapeutic agent in lung tumors. This compound significantly induced the apoptosis of A549 and PC9 lung cancer cells in a dose-dependent manner via the mitochondrion-mediated and caspase-dependent apoptotic pathway [41]. Simultaneously, an increase in cytochrome C and an enhancement of caspase-9 and caspase-3 activities were observed in A549 and PC9 lung cancer cells. In vitro inhibition of proliferation of lung cancer cell lines H1299/M and PA/M required a high concentration of amygdalin [42]. However, at a lower concentration of amygdalin, it was observed that the invasion and migration abilities of H1299/M PA/M cancer cells were significantly inhibited [42]. Thus, it was suggested that amygdalin is likely to have antimetastatic activity, inducing apoptosis and inhibiting the proliferation of lung cancer cells [42].

#### 3.2.2. Breast Cancer

Amygdalin has been shown to induce apoptosis and inhibit the adhesion of breast cancer cells by increasing the level of pro-apoptotic Bax proteins and caspase-3 activity and decreasing the level of the anti-apoptotic Bcl-2 protein [20]. Significantly, in both MCF-7 and MDA-MB-231 breast cancer cells, amygdalin significantly increased apoptosis by suppressing cell proliferation and improving radiotherapy efficiency through induction of cell cycle arrest (in G1 and sub-G1 cell cycle stages) [11]. Amygdalin was also found to decrease the migration of MDA-MB-231 cells more than MCF-7 cells [43]. Furthermore, the inhibition of proteolytic enzymes was suggested to promote the activation of apoptotic events in MCF-7 breast cancer cells [44]. In addition, amygdalin was shown to increase Bax and decrease Bcl-2 expression in SK-BR-3 and MCF-7 breast cancer cells. However, compared with the amygdalin–ZHER2 affibody conjugate, the effect on Bax and Bcl-2 expression in SK-BR-3 cells was stronger than that in MCF-7 cells [45]. Τhe ability of amygdalin to reduce MCF-7 and T47D human breast cancer cell growth in a concentration-dependent manner by stimulating malondialdehyde (MDA) and oxidized glutathione production was also demonstrated. Moreover, considerable reductions of total glutathione levels and glutathione reductase activity in breast cancer cells were observed [46].

#### 3.2.3. Prostate Cancer

Amygdalin dose-dependently inhibited tumor growth and reduced tumor clones in prostate cancer cell lines by inhibiting the G0/G1 phase [47]. Moreover, inhibition of prostate cancer cell growth and tumor growth by amygdalin were evident, revealing a function of the metabolic enzymes betaglucosidase (β-glucosidase) and rhodanese in regulating the anticancer activity of amygdalin in vivo [10]. The activation of amygdalin by β-glucosidase could be considered an enzyme/drug therapy strategy that may be a promising new approach for the targeted treatment of prostate cancer [48]. The exposure of certain prostate cancer cells, such as DU-145, to amygdalin was also found to inhibit metastatic spread promoted by α6 integrin [49]. 

#### 3.2.4. Colorectal Cancer

In alternative and traditional medicine, amygdalin is commonly used for the prevention and treatment of colorectal tumor malignancies [50]. The anticancer effect of amygdalin on colorectal cancer cells, for instance in human SNU-C4 colorectal cancer cells, has been found to be promoted by reducing the expression of cell-cycle-related genes [51]. Colon cancer cells were reported to be more sensitive to the effect of amygdalin compared to normal cells due to their higher concentration of β-glucosidase and lower levels of the liver enzyme rhodanese, which can convert cyanide to the relatively harmless compound thiocyanate [52]. 

#### 3.2.5. Cervical Cancer

Amygdalin has been documented to significantly inhibit the proliferative activity of HeLa cervical cancer cells [19]. The anti-apoptotic protein Bcl-2 was downregulated and the pro-apoptotic Bax was upregulated in HeLa cells treated with amygdalin [22]. Moreover, the Bax-to-Bcl-2 ratio and caspase-3 activity were increased by amygdalin treatment in HeLa cells, reinforcing the apoptotic effect of amygdalin on cervical cancer cells [22,53].

#### 3.2.6. Gastrointestinal Cancer

Amygdalin has been demonstrated to stimulate the apoptotic process by upregulating caspase-3 expression and downregulating Bcl-2 expression, as well as inhibiting HepG2 and EAC hepatocellular cancer cell proliferation and upregulating Beclin-1 expression [54]. It is noteworthy that the combination of amygdalin with metformin exerted a promising effect when compared to amygdalin alone; their combination was more cytotoxic, showing a greater ability to induce apoptosis and arrest the cell cycle in hepatocellular cancer cells [55]. In addition to this combination, the activity of amygdalin with zinc has also been shown to produce an enhanced apoptotic effect in the treatment of HepG2 compared to the effect of amygdalin without zinc [56].

#### 3.2.7. Other Tumor Malignancies

The inhibitory effect of amygdalin on the growth and differentiation markers E- and N-cadherin in renal cell carcinoma (RCC) cells was also demonstrated via the application of 10 g/mL amygdalin to the RCC cell lines A498, Caki-1, and KTC-26 for a period of 24 h or 2 weeks in vitro [35]. The investigation of amygdalin’s (1.25–10 mg/mL) impact on several bladder cancer cell lines (UMUC-3, RT112, and TCCSUP) also showed positive results [57]. The most remarkable impacts of amygdalin have been attributed to the cdk2–cyclin A axis. Studies on siRNA knockdown have shown a positive relationship with cdk2/cyclin. Amygdalin has also been found to inhibit tumor development via downregulation of cdk2 and cyclin [57]. In contrast, colony-forming cells from leukemic cell lines and normal bone marrow were relatively tolerant to amygdalin and its metabolites in vitro. Although an increasing rate of apoptosis was observed, there was no selective destruction between human leukemic cell lines and normal bone marrow cells [58].

**Table 1:** Studies evaluating the anticancer effects and the anticancer molecular mechanisms of amygdalin.

Study Design	Dosage of Amygdalin	Action of Amygdalin	References
Animal study (in vitro and in vivo) N = 18	1.25, 2.5, 5, 10, and 20 mg/mL, intraperitoneal injection, cytotoxic, ↓ viability of HeLa cells in a dose-dependent manner.	↑ Apoptosis by ↑ activity of caspase-3, ↑ Bax, and ↓ Bcl-2 protein in cervical cancer cells (HeLa).	[22]
Test-tube lab on human cells N = (5 × 10^5^ cells/well)	1, 2, 5, 10, and 20 mg/mL, cytotoxic to HL-60 cells (6.4 mg/mL) in the presence of 250 nM β-glucosidase.	↑ Apoptosis in human promyelocytic leukemia cells (HL-60, ATCC CCL240).	[58]
Clinical trial N = 178 patients	3 g (intravenous administration) and 0.5 g (oral use), symptoms of cyanide toxicity or by blood cyanide levels approaching the lethal range.	No substantive benefits in terms of cure, improvement or stabilization of cancer, improvement of symptoms related to cancer, or extension of life span.	[59]
Test-tube lab in vitro N = (1 × 10^5^ cells/mL)	10 mg/mL, no cytotoxic effects were detected in vitro.	↓ Tumor cell growth, ↓ P19 and P27 expression in renal cancer cells (Caki-1, KTC-26, and A498).	[35]
Animal study (in vitro and in vivo) N = 20	40 mg/kg and 80 mg/kg amygdalin in saline, intragastrically, no significant toxic effects were observed.	↑ Apoptosis and proliferation inhibition of lung cancer cells (A549, PC9, H1299, and PA).	[42]
Animal study (in vivo), N = 30	10 mg/kg (nano-extract of *P. persica*) and 15 mg/kg (nano-extract solution of *P. persica* seed extract).	No differences in the histological structure of the liver, central veins, and hepatic chords in rat liver tissues.	[60]
Test-tube lab on human cells (in vitro) N = (2 × 10^4^ cells/cm^2^)	4, 8, 16, 32, and 65 mmol/L of an amygdalin solution, different cytotoxic effects between the two cell lines.	↓ Oxidative stress in human breast cancer cell lines (MCF-7 and T47D).	[46]
Test-tube lab (in vitro) N = (1 × 10^5^ cells/mL)	10, 20, and 40 mg/mL, incubation in serum-free medium.	↑ Apoptosis, ↑ Bax proteins, and caspase-3 activity, ↓ Bcl-2 protein in breast cancer cells (Hs578T, MCF-7, and MDA-MB-231) Data demonstrated that amygdalin exerted cytotoxic effect.	[43,44,45,46]
Test-tube lab on human cells, N = (1 × 10^5^ cells/well)	0.25, 0.5, 2.5, and 5 mg/mL for 24 h, no cytotoxic effects were detected.	Apoptosis-related genes downregulated by amygdalin in human colon cancer cells (SNU-C4).	[51]
Test-tube lab on human cells, N = (2 × 10^4^ cells/cm^2^)	0.01 mg/mL, 0.1 mg/mL, 1 mg/L, and 10 mg/mL, amygdalin exerted a dose-dependent cytotoxic effect	↓ Tumor cell growth, ↑ apoptosis, ↓ metastatic spread by α6 integrin, ↑ Bax, and ↓ Bcl-2 protein in prostate cancer cells (DU145 and LNCaP).	[47,48,49]
Clinical trial N = 6 patients	4.5 g/sq m/day (intravenously for 21 days) and 0.5 g three times daily, no evidence of toxic reaction.	Slight ↑ of thiocyanate levels in plasma in 50% of patients with malignant disease.	[32]
N = 21 healthy volunteers: Chinese adults	Single doses (3, 6, and 9 g) and multiple doses (6 g, once daily) were administrated intravenously and the safety profiles were evaluated.	No adverse effects and a good tolerability were observed.	[61]
Test-tube lab, N = (5 × 10^4^ cells/mL)	5, 10, 15, or 20 μg/mL, no toxic effects were reported.	↓ Bcl-2, ↑ caspase-3 expression in hepatocellular cancer cells (HepG2 and EAC).	[54,55,56]
Test-tube lab on human cells (in vitro)	3, 6, and 9 μg/mL, slight ↓ of cell survival (80%), suggesting a weak genotoxic effect.	Anticancer effect in colorectal cancer cells (HepG2, HT-29, BALB/3T3, clone A31, and BJ).	[50]
Animal study (in vivo, five groups of nude mice)	0.5 mL/daily, no toxic effects were reported.	No change of tumor volume in prostate cancer cell lines (PC3).	[10]
Test-tube lab (in vitro) N = (1 × 10^4^ cells/mL)	1.25–10 mg/mL, no signs of toxicity were shown.	↓ Bladder cancer cell growth by ↓ cyclin A, CKD2, and P27 protein.	[57]
In vitro	Nanoformulation of amygdalin by β-CD	β-CD-Amygdalin exerted greater effects than amygdalin. The nanoformulation of amygdalin with β-CD ↑ breast cancer cells mitigation, gene mitigation, and oxidative stress.	[62]
In vitro	Silver nanoparticles encapsulated with amygdalin, in breast cancer cell lines, cross-linking to microcapsules charged with chitosan.	Controlled release of amygdalin led to overcoming of low cytotoxic effects in high doses, showing anticancer activity.	[63]
In vitro	Alginate–chitosan nanoparticles (were used as drug administration for amygdalin encapsulation and its delivery to tumor cells lines (H1299).	ACNPs demonstrated greater antitumor effect on H1299 cell lines than free amygdalin, suggesting greater cellular uptake of nanoparticle compound.	[64]
In vitro	Nanoparticles demonstrated sustained amygdalin–folic acid release properties and obvious selectivity to cells	Cell cycle blocking. Malignancy growth suppressing. ↓ Iron levels, and mitogen-activated protein kinases (MAPK/P38) by ↓ iron levels and MAPK/P38. Inhibition of differentiation complex (CD4 and CD80) expression. ↓ Transformation of growth factor beta interleukin-6, interferon-gamma, interleukin-2, and vascular endothelial growth factor (VEGF) expression in the signaling pathway. Modulation of gene expression of CD8 and the natural killer group 2D. ↓ Proliferation of breast cancer cells.	[11]

↑: increase; ↓: decrease; Bcl-2: B-cell lymphoma 2; CKD2: cyclin-dependent kinase 2; P27: cyclin-dependent kinase inhibitor 1B; P19: RNA silencing suppressor p19; βCD: β-cyclodextrin; ACNPs: alginate chytosan nanoparticles.

### 3.3. Toxicity of Amygdalin 

Excessive consumption of amygdalin can lead to poisonous effects (more than 1 mg/L cyanide in the blood). Amygdalin is converted into glucose, benzaldehyde, and hydrogen cyanide by an endogenous enzyme (β-glucosidase) when fruit pits are crushed. More analytically, when HCN is released, cytochrome oxidase C can react with the iron ion. This can induce the formation of metal-ion complexes which lyse cells and inhibit ATP synthesis [65]. 

Amygdalin has been reported to exert toxic effects when ingested with supplements. Oral intake of 500 mg amygdalin might release 30 mg of cyanide [66]. Cyanide toxicity can be life-threatening due to the decrease of mitochondrial oxygen utilization, leading to cell death. Cancer cells lack rodhanase, an enzyme which acts as a detoxifying agent by binding iron sulfur centers on cell membranes and converts HCN into a less toxic metabolite, thiocyanate. However, following parenteral administration of amygdalin/laetrile by injection, more than 80% of thiocyanate was detected in rats’ and rabbits’ urine [66]. The adverse side effects of cyanide toxicity include tachycardia, confusion, nausea, headache, and, more severely, neuromyopathy, cyanosis, coma, convulsions, and death [67].

Over recent decades, several in vitro and in vivo studies have been performed, using single or multiple doses and different forms of amygdalin administration (intravenous and intramuscular), that showed no HCN formation, highlighting the crucial role of the gut in human body physiology after substance consumption. The anaerobic bacterial phyla existing in the gut present a high β-glucosidase activity, which is needed for amygdalin to hydrolyze HCN. Nevertheless, HCN toxicity has been found to exist under certain circumstances. In some cases, toxicity derived from the ingestion of variable doses of amygdalin and there were no HCN side effects associated with high doses. Several factors, including probiotic or prebiotic consumption, diet, and age, may alter the gut consortium, which is responsible for the conditions under which toxicity occurs. Notably, serious reactions were not reported for a dose of 3 g orally administrated amygdalin in patients with cancer who were seeking alternative therapies. The minimum lethal dose of amygdalin for an adult is 50 mg or 0.5–3.5 mg/kg of body mass. However, the interaction with concomitant consumption of vitamin C seems to activate its adverse side effects, while vitamin B12 and sodium diosulfate solution have been used as antidotes without adverse side effects [65].

### 3.4. Amygdalin/Laetrile Clinical Studies in Human Tumor Malignancies in the 20th Century

Several studies have demonstrated the anticancer activity of amygdalin and its therapeutic use for cancer treatment and pain relief [30,68]. Although the scientific evidence for the anticancer effect of amygdalin based on clinical trials is limited, there have been some trials examining the effect of amygdalin against tumor malignancies in humans. Several clinical trials of amygdalin/laetrile have been considered over the years [32]. In 1980, the Research Drug Branch of the National Cancer Institute (NCI) announced that approximately 200 cancer patients “for whom no other treatment has been effective” were planned to receive the chemical, a special diet, and supplemental vitamins (see “National Cancer Institute begins laetrile clinical trial”, 1980) [69]. Two clinical trials in the field of laetrile in human cancer were conducted over the next two years. The first of these two clinical trials was performed in 1981 and it was conducted on six patients with advanced cancer [32]. Amygdalin was administered both intravenously and orally for a duration of 21 days, with no evidence of toxic reactions. These findings were in accordance with previous observations of a patient after self-administration of laetrile [70]. In 1982, another clinical trial was performed with a total number of 178 patients with cancer who received an amygdalin treatment plus a “metabolic therapy” [59]. No substantial benefits were observed in terms of cure, improvement or stabilization of cancer, improvement of symptoms related to cancer, or extension of life span. The hazards of amygdalin therapy were evidenced in several patients by symptoms of cyanide toxicity or by blood cyanide levels approaching the lethal range [59,69,70]. However, it should be noted that these clinical trials were performed over 40 years ago and should now be considered outdated, highlighting the need for novel clinical trials carried out involving the administration of amygdalin with diverse pharmaceutical formulations that could be more tolerable to and acceptable by the human body, presenting more bioactive efficiency and nontoxic effects.

### 3.5. Nanoparticles and Amygdalin in the 21st Century

Nanoparticles are considered a promising method in biotechnology for drug delivery and the treatment of human tumor malignancies while avoiding toxicity. Several studies in human cancer cell lines have demonstrated positive results concerning amygdalin metabolism without side effects. As already mentioned, amygdalin, despite its anticancer actions, has been faced controversy because of the cyanide release. Sohail and Abbas investigated alginate–chitosan nanoparticles (ACNPs) as a mode of drug administration via amygdalin’s encapsulation and delivery to tumor cells (H1299) [64]. The nanoparticles showed stable drug release over a ten-hour period and significant swelling rates in slightly acidic and neutral environments. ACNPs were shown to have a greater antitumor effect on H1299 cell lines than free amygdalin, suggesting greater cellular uptake of the compound encapsulated in nanoparticles. In this regard, biomimetic and biocompatible balginate–chitosan nanoparticles could be applied as an advantageous drug delivery system for prolonged and controlled delivery of amygdalin with enhanced cytotoxic activity against tumor cells, while simultaneously protecting normal human tissues and healthy cells [64].

Silver nanoparticles encapsulating amygdalin and cross-linking to microcapsules charged with chitosan have also been explored in breast cancer cell lines. An anticancer response was also observed in line with the controlled release of amygdalin due to chitosan conjunction, overcoming low cytotoxic effects at high doses [63].

Additionally, nanoparticles demonstrated sustained amygdalin–folic acid release properties and apparent selectivity to cells by suppressing tumor growth. At the same time, they were found to improve radiotherapy efficiency by enhancing apoptosis, blocking the cell cycle, and reducing the proliferation of breast cancer cells by downregulating iron levels and mitogen-activated protein kinases (MAPK/P38). Amygdalin–folic acid was also shown to inhibit the differentiation of CD4 and CD80 complex expression, inducing suppression of transformation of growth factor beta (TGF-beta)/interleukin-6, (IL-2)/interferon-gamma, (INF-g)/interleukin-2, and (IL-2)/vascular endothelial growth factor (VEGF) expression at the signaling pathway, while simultaneously modulating the gene expression of CD8 and the natural killer group 2D [11].

Mosayyebi and colleagues nanoformulated amygdalin with β-cyclodextrin in order to investigate the increase of its action against MCF-7 cell line migration, apoptosis, and migration of genes. The nanoformulated amygdalin showed a greater effect on tumor cells than amygdalin alone [62]. 

### 3.6. Nutritional Supplements with Amygdalin for Cancer Treatment

Amygdalin, laetrile, or vitamin B17 has been claimed as a treatment for various diseases, especially tumor malignancies, since 1845 [33]. However, in 1982, there was a perception that amygdalin might be a toxic drug and not effective in cancer treatment [59]. Recent theoretical and practical developments have revealed that amygdalin may produce beneficial effects for cancer patients [28,62,63]. Amygdalin has been used to treat cancer both as a single agent and in combination with metabolic therapy. Therefore, it is worth mentioning that toxicity from vitamin supplements is not a rare occurrence and amygdalin is recommended to patients as a dietary supplement for cancer, wherein high doses are proposed [71]. Amygdalin tablets and capsules are currently marketed as a natural dietary supplement under the misnomer laetrile or the questionable name “vitamin B17” [72].

Certain case report studies have demonstrated rebound metabolic acidosis following massive amygdalin overdose and life-threatening cyanide toxicity, including nephrogenic diabetes insipidus [73,74,75,76]. Amygdalin toxicity can be caused by the poisonous composite product of benzaldehyde and cyanide after oral ingestion [35]. Both toxicologists and nephrologists should be aware of the potential of this “vitamin” to cause cyanide poisoning [77]. Furthermore, there is currently a serious concern that natural dietary supplements are not subjected to rigorous analytical and clinical trials. Based on the European Union Regulation ((EC) No. 178/2002) concerning general food legislation, dietary supplements are considered foods and not drugs [78]. According to the above data suggesting that the clinical use of amygdalin dietary supplements may be accompanied by adverse side effects, the risk–benefit balance is not therefore favorable for amygdalin in this respect [78,79]. Moreover, amygdalin is incorrectly referred to as vitamin B17; the compound is not a vitamin [36].

## 4. Discussion

In alternative medicine, amygdalin has been considered an anticancer treatment for several decades without rigorous scientific support for its efficacy and safety. Several case studies have highlighted the risk of poorly regulated supplements [79]. Recent in vitro studies have demonstrated that amygdalin can exert anticancer activity by affecting the cell cycle, promoting apoptosis and cytotoxicity, and modulating immune response [29,80,81]. However, clinical trials have shown that metabolites of amygdalin can be turned into hydrocyanic acid and that hydrocyanic acid accumulation, with the passage of time, may result in an adverse toxic effect in humans [82].

Moreover, the currently available studies have some limitations. Only a few in vivo animal studies have been performed to date. Additionally, the studies’ results are inconsistent, perhaps because of the heterogeneity of their method design. Dose, form of the substance, type of administration, the lack of RCTs in humans, and the lack of phase III and IV clinical trials are considerable limitations to the recommendation of amygdalin administration in cancer prevention and/or treatment in clinical practice. Thus, a strength of our review article concerns the identification of a literature gap concerning the performance of clinical studies of amygdalin. On the other hand, there is still a lack of reliable data concerning the bioavailability of amygdalin and its corresponding concentration levels in the blood circulation. Accordingly, no data exist concerning whether amygdalin may be used as a co-treatment together with other chemotherapeutics.

A significant research scientific gap between the end of the 20th century and the beginning of the 21st century has emerged as limited research activity has been performed to date. Moreover, most studies supporting the anticancer effects of amygdalin have been performed in various cancer cell lines in vitro. Thus, it is reasonable to suggest that its anticancer effects cannot be extrapolated to humans. Conflicting outcomes from in vitro and in vivo studies [41,51,60,83,84,85,86] and from a few clinical trials [61] further highlight the need for additional research in the field of cancer therapy, especially related to the investigation of a new, nontoxic formula of amygdalin, and bearing in mind the role of nanotechnology in the current era of biomedical science. Overall, there is a strong demand for further in vivo animal studies as well as clinical human studies to explore the potential prevention and/or treatment efficiency of amygdalin against cancer disease development and progression. In addition, the discrepancies found in some clinical trials may be ascribed to low sample sizes as well as to the different personalized characteristics of the enrolled individuals, highlighting the need to perform well-designed clinical studies with adequate sample sizes in the future.

The Cochrane Database of Systematic Reviews claimed in 2015 that laetrile or amygdalin exerts beneficial effects in cancer patients, which is not currently supported by scientifically sound clinical data [28]. The above report documented that there is a considerable risk of serious adverse side effects from cyanide poisoning after laetrile or amygdalin administration, especially after oral ingestion [28]. Thus, the risk–benefit balance of laetrile or amygdalin as a treatment for cancer remains unambiguously questionable [28]. However, much research has been performed since 2015. Moreover, the National Cancer Institute has reported that the incidence of cyanide poisoning is much higher when laetrile is taken orally because intestinal bacteria and some commonly eaten plants contain enzymes (beta-glucosidases) that activate the release of cyanide after laetrile ingestion [87]. 

Finally, by effectively applying recent developments in drug discovery processes, amygdalin could be used as a lead compound to synthesize and develop more bioactive amygdalin-related analogues with higher efficiency and target selectivity, and with reduced adverse side effects and improved oral bioavailability. For example, machine learning techniques have revolutionized the field of structure-based drug design in recent years [88]. Artificial intelligence approaches to speed up and prevent failures in the drug discovery pipeline could also be applied in the case of amygdalin [89]. Beyond machine learning and artificial intelligence methods, quantum computing is another significant advance in technology in generative chemistry and drug discovery processes, which researchers may exploit in the case of amygdalin [90]. Late-stage functionalization also presents novel challenges for the introduction of new chemical moiety groups, such as amygdalin and its future potential synthetic analogues, toward the end of a synthetic sequence, which means that new molecules that could be rapidly accessed without laborious de novo chemical synthesis [91]. This specific approach may offer benefits such as efficient access to diverse libraries to explore structure–activity relationships and the improvement of physicochemical and pharmacokinetic properties [91].

Computer-aided drug discovery can also achieve rapid identification of highly diverse, potent, target-selective, and drug-like ligands to proteins, presenting new opportunities for the cost-effective development of safer and more effective small-molecule treatments such as amygdalin [92]. Lipophilicity and biomimetic properties also have important distinct and overlapping roles in supporting the drug discovery process, mainly by increasing drug candidates’ oral bioavailability and considerably reducing their potential adverse side effects [93,94]. Lipophilicity is uniquely valuable in early drug design for library screening and for the identification of promising compounds to start with, while biomimetic properties are useful for the experimentally based evaluation of absorption, distribution, metabolism, and excretion (ADME) properties of synthesized novel compounds, supporting the prioritization of drug candidates and guiding further synthesis; these approaches could be applied in the case of amygdalin to obtain new amygdalin synthetic analogues with increased oral bioavailability and reduced adverse side effects [93,94].

In support of the above considerations, double-docking and molecular dynamics simulations have recently been applied to develop novel approaches to explain the effect of amygdalin on the dynamic behaviors of the Bcl-2/BAX complex, caspase-3, and PARP-1 [95]. These molecular targets may play determinant roles in apoptotic pathways and could be considered potential therapeutic targets for cancer treatment [95]. Overall, these computational observations may be considered good evidence for rejection of the belief that the cyano-group of amygdalin, which is the major group responsible for the anticancer activities of amygdalin [95], can be substituted with another chemical moiety with lower adverse effects [96,97]. Additionally, computational results have confirmed that amygdalin has a unique structure and could be considered a reference compound for drug designers to develop new molecules with similar effective anticancer chemical structures but with lower adverse side effects [95,96,97].

## 5. Conclusions

Current there are several lines of in vitro evidence suggesting that amygdalin and its synthetic analogue, laetrile, possess anticancer properties, and previous and upcoming in vivo animal studies appear to confirm their anticancer properties. However, there are certain emergent and severe issues related to their toxicity due to their cyano-moiety as well as their poor oral bioavailability. In this regard, new technologies in drug development should be applied effectively to minimize their adverse side effects while also enhancing their oral bioavailability. In this regard, medicinal chemists should be focused on the laboratory synthesis of chemical analogues which could maintain the anticancer activity of amygdalin while reducing its adverse side effects. Nanoparticle technology seems promising to increase the bioavailability and the anticancer activity of amygdalin while simultaneously decreasing its toxic effects. However, there is a significant gap in the literature concerning the performance of clinical trials to explore its anticancer activity in humans and to ensure that amygdalin is safe before its introduction in clinical practice. A combination of nanoparticle technology with the use of novel and safer synthetic analogues of amygdalin may increase the efficiency of this natural compound and represent a new therapeutic strategy for the treatment of cancer.

## Data Availability

The data are available upon request to the corresponding author.
